# Supplementary material for: Ion-specific binding of cations to the carboxylate and of anions to the amide of alanylalanine
Source: Commun Chem. 2022 Dec 20;5:173. doi: 10.1038/s42004-022-00789-y (PMC9814750; doi:10.1038/s42004-022-00789-y)
Supplement: Supplementary file 2 — Supplementary Information [file 42004_2022_789_MOESM2_ESM.pdf]

SUPPLEMENTARY INFORMATION

for

Ion-specific binding of cations to the carboxylate and of anions to the amide of alanylalanine

Carola Sophie Krevert [1], Lucas Gunkel [1], Constantin Haese [2] and Johannes Hunger [1]

[1] Department of Molecular Spectroscopy, Max Planck Institute for Polymer Research, Ackermannweg 10, 55128 Mainz, Germany

[2] Department of Molecular Electronics, Max Planck Institute for Polymer Research, Ackermannweg 10, 55128 Mainz, Germany

[*] Email: hunger@mpip-mainz.mpg.de

Supplementary Discussion 1: DR Experiments

Aqueous solutions of 2Ala

In the main text we only qualitatively discuss the relaxation parameters and the derived effective dipole moments from the dielectric experiments on aqueous 2Ala solutions. Here we discuss the obtained parameters in more detail. For aqueous 2Ala solutions the increasing relaxation strength, S_{2Ala}, (Figure 2b, main text) with increasing peptide concentration, c_{2Ala}, can be quantitatively analyzed: S_{2Ala} scales with the concentration of the dipolar species and its squared dipole moment, $S \propto c \cdot {\mu_{\mathrm{eff}}}^2$. The increase in S_{2Ala} can therefore stem from both, increasing c_{2Ala} or an increasing effective dipole moment, μ_{eff}. Changes in μ_{eff} can result from conformational changes or aggregation (e.g. anti-parallel dipole correlations of 2Ala[1]). In order to distinguish between the increase in S_{2Ala} due to increasing c_{2Ala} and conformational changes or aggregation, we calculate μ_{eff} according to:[2,3]

$$\frac{2\,\varepsilon_{\mathrm{S}}+1}{\varepsilon_{\mathrm{S}}} \cdot S = \frac{N_{\mathrm{A}}\, c}{k_{\mathrm{B}}\, T\, \varepsilon_0} \cdot \mu_{\mathrm{eff}}^2 \tag{S1}$$

Here, N_A is Avogadro's constant, k_B the Boltzmann constant, ε_S the static permittivity, ε_0 the vacuum permittivity, and T the thermodynamic temperature.

The results are shown in Figure 2c (main text). The effective dipole moment of 2Ala stays virtually constant at concentrations ranging from 0.05 M to 0.25 M. The insensitivity to concentration indicates that the changes in amplitude solely result from the increase in concentration and concentration-dependent changes in the 2Ala conformation or aggregation can be ruled out.

With increasing concentration of 2Ala, both the dispersion in $\varepsilon'(\nu)$ and the peak in $\varepsilon''(\nu)$ due to the peptide at ~ 1 GHz slightly shifts to lower frequencies, indicative of a retardation of the peptide dynamics: the rotational relaxation time τ_{2Ala} somewhat increases from 165 to 186 ps (Supplementary Figure 1c) with increasing concentration. In general, the relaxation time scales with the microscopic viscosity η' and the effective volume V_{eff}.[4] Given that we have excluded conformational changes based on the discussion of the dipole moments, changes in the effective volume can be neglected. Thus, the increase of the relaxation time with increasing concentration can be ascribed to an increase in microscopic viscosity, [5] in line with results from other DR studies on small peptides. [6,7]

The water amplitude S_{H2O} slightly decreases with increasing peptide concentration (from 72 to 70, see Supplementary Figure 1a). This decrease can be explained by the sample compositions: With increasing peptide concentration, peptide molecules replace water, thus reducing the concentration of water. The rotational relaxation time τ_{H2O} increases from 8.3 ps (neat water) to 8.8 ps at c_{2Ala} = 0.25 M. This increase could be assigned to increasing viscosity and/or slower dynamics in the peptide's hydration shell,[6] in line with previous reports.[6,8]

Ternary water+2Ala+salt solutions

Figure 3a (main text) shows the concentration dependent spectra of aqueous 2Ala-LiCl solutions. The spectra for other ternary samples (with added GdmCl, KCl, KI, or KSCN) are shown in Supplementary Figure 2. We fit the same Cole-Cole + Debye model (Equation 1 in the main manuscript) to the spectra of all ternary samples. The corresponding fit parameters are illustrated in Supplementary Figure 3.

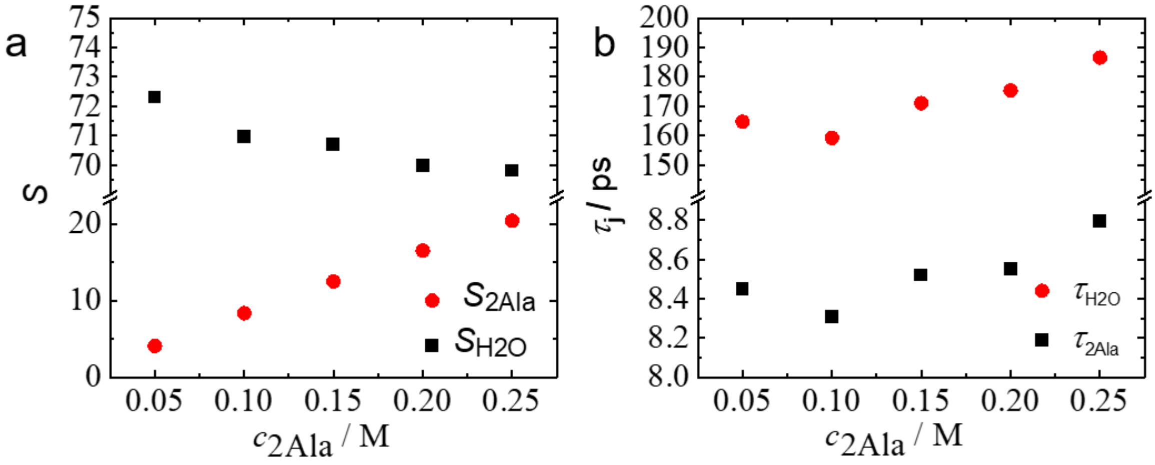

Supplementary Figure 1: Relaxation parameters as obtained for aqueous solutions of 2Ala. a) Amplitudes S_{2Ala} and S_{H2O} as a function of the concentration of 2Ala. b) Ratio of water amplitude and water concentration. c) Rotational relaxation times τ_{2Ala} and τ_{H2O}.

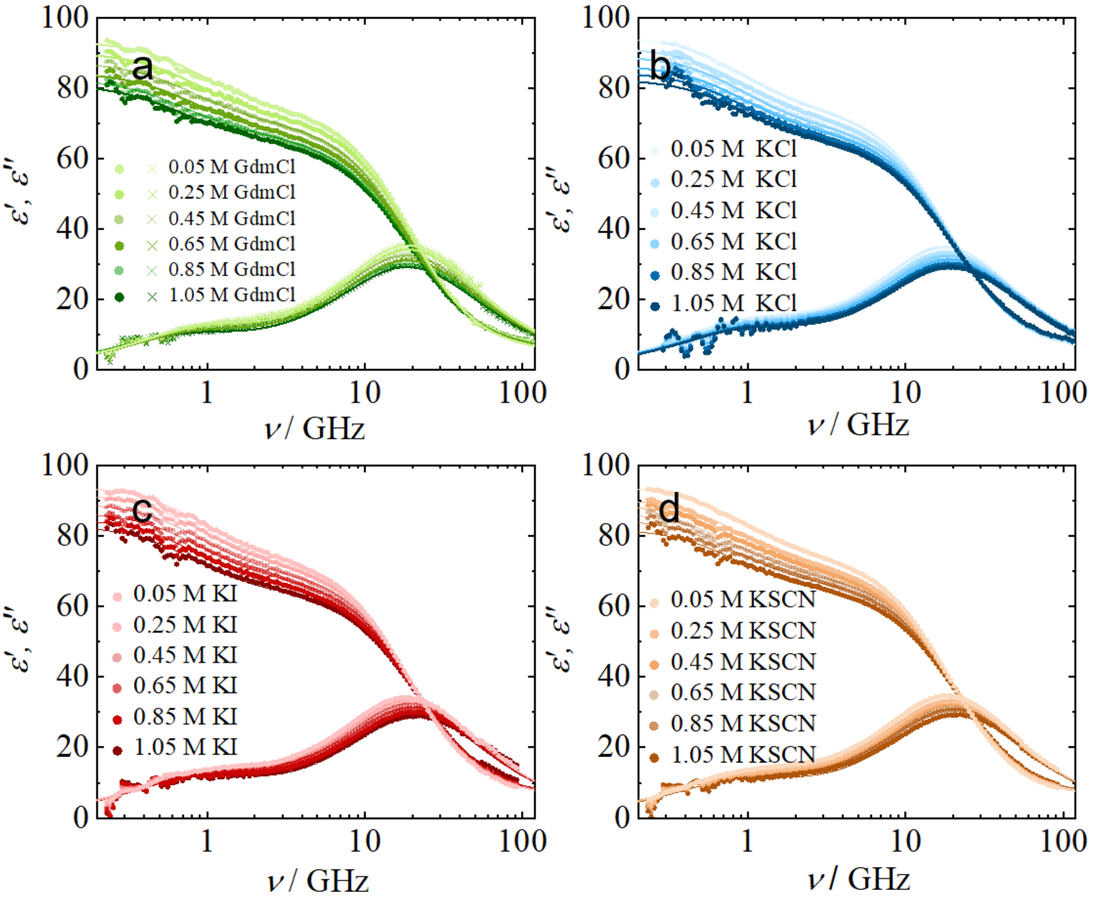

Supplementary Figure 2: DR spectra of aqueous (250 mM) 2Ala solutions with increasing concentration of a) GdmCl, b) KCl, c) KI, and d) KSCN. Symbols show experimental dielectric permittivity (ε') and dielectric loss (ε'') spectra. Solid lines show fits of eq. 1 (main manuscript) to the experimental data. Note that the conductivity contributions (last term of eq. 1 in the main manuscript) have been subtracted for visual clarity.

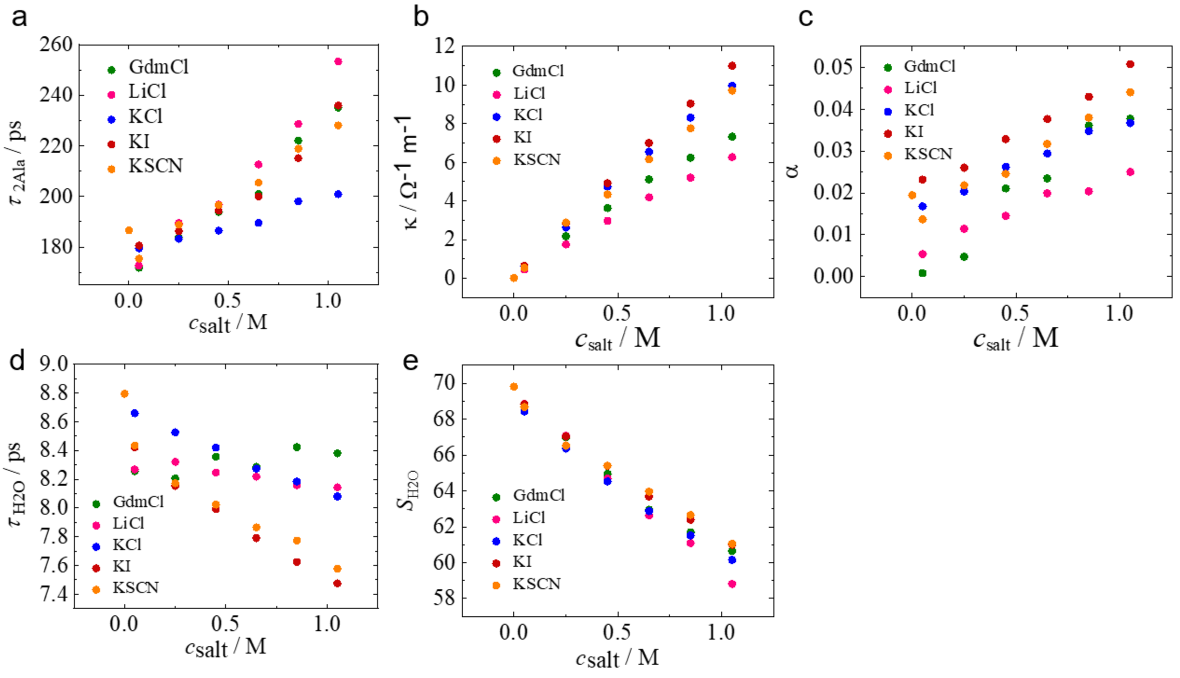

Supplementary Figure 3: Fit parameters obtained from fitting equation 1 (main text) to the experimental dielectric spectra of aqueous salt + 2Ala solutions.

Supplementary Discussion 2: NMR Experiments

Supplementary Figure 4 shows the chemical structure of 2Ala with the corresponding labels of the C and H nuclei, used for the assignment of the NMR signals.

In Figure 4a (main text), the ^{1}H-NMR spectrum for a 0.25 M 2Ala solution is shown: ^{1}H-NMR (in H_2O (DMSO-d5h1 capillary), 400 MHz): δ/ppm 8.04 (s, 1H, H-5), 3.88 (q, $J^3{}_{HH}$ = 7 Hz, 1H, H-4), 3.82 (q, $J^3{}_{HH}$ = 7 Hz, 1H, H-3), 1.29 (d, $J^3{}_{HH}$ = 7 Hz, 3H, H-1), 1.10 (d, $J^3{}_{HH}$ = 7 Hz, 3H, H-2). This assignment of the proton signals (Figure 4 main text and Supplementary Figure 5 for 2Ala in H_2O) and carbon signals (Supplementary Figure 6) is based on 2D NMR spectra, which are displayed in Supplementary Figure 7 (^{1}H-^{13}C-HSQC) and Supplementary Figure 8 (^{1}H-^{13}C-HMBC). The assignment of proton signal H-5 to solely the NH proton is based on integrated signal intensities for the spectrum of 2Ala in H_2O: the integrals of the methyl group signals (H-1, H-2) are approximately three times higher than the integral of the H-5 signal (Supplementary Figure 5).

In the main text, we show the variation of the chemical shift of all detected protons only for KI (Figure 5a, main manuscript). To illustrate the variation of the chemical shift from the raw spectra, we show the ^{1}H NMR spectra of 2Ala+KI solutions for different salt concentrations in Supplementary Figure 9. These spectra are dominated by the water signal, which is shifted up-field with increasing salt concentration (analogously to the medium effect discussed in the main manuscript). The proton signals of 2Ala are readily resolved (Supplementary Figure 9). The variation for other salts is displayed in Supplementary Figure 10 for a) GdmCl, b) LiCl, c) KCl, and d) KSCN. To exclude a contribution of a salt-induced aggregation of 2Ala to the observed variation of the chemical shift, we have performed experiments for samples containing 2Ala and KI with a reduced concentration of 2Ala (50 mM). The data in Supplementary Figure 11 demonstrate that the results at 50 mM 2Ala nearly quantitatively agree with the results obtained at 250 mM 2Ala, suggesting that a potential salt-induced aggregation of 2Ala does not affect our observations.

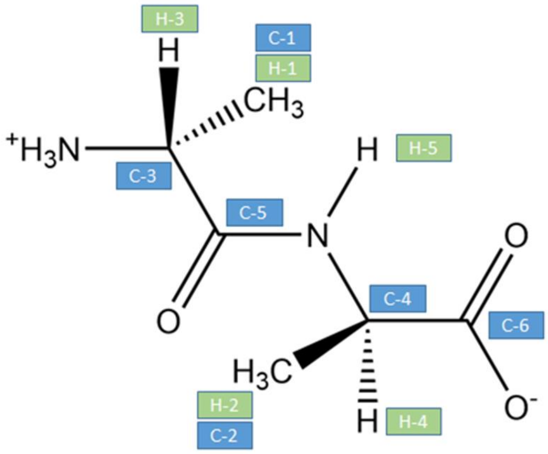

Supplementary Figure 4: Chemical structure of 2Ala with the corresponding labels for C and H nuclei as used in the discussion of the NMR experiments.

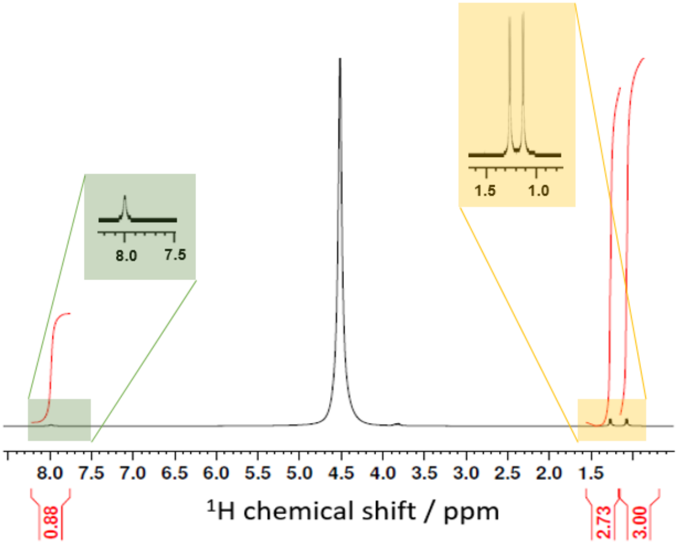

Supplementary Figure 5: ^{1}H-NMR spectrum of 2Ala + 0.65 M KCl in H_2O (DMSO-d5h1 capillary included). Integrals for methyl groups (at ≈ 1 ppm) relative to the NH proton signal at ≈ 8 ppm obey an approximate 3:1 ratio.

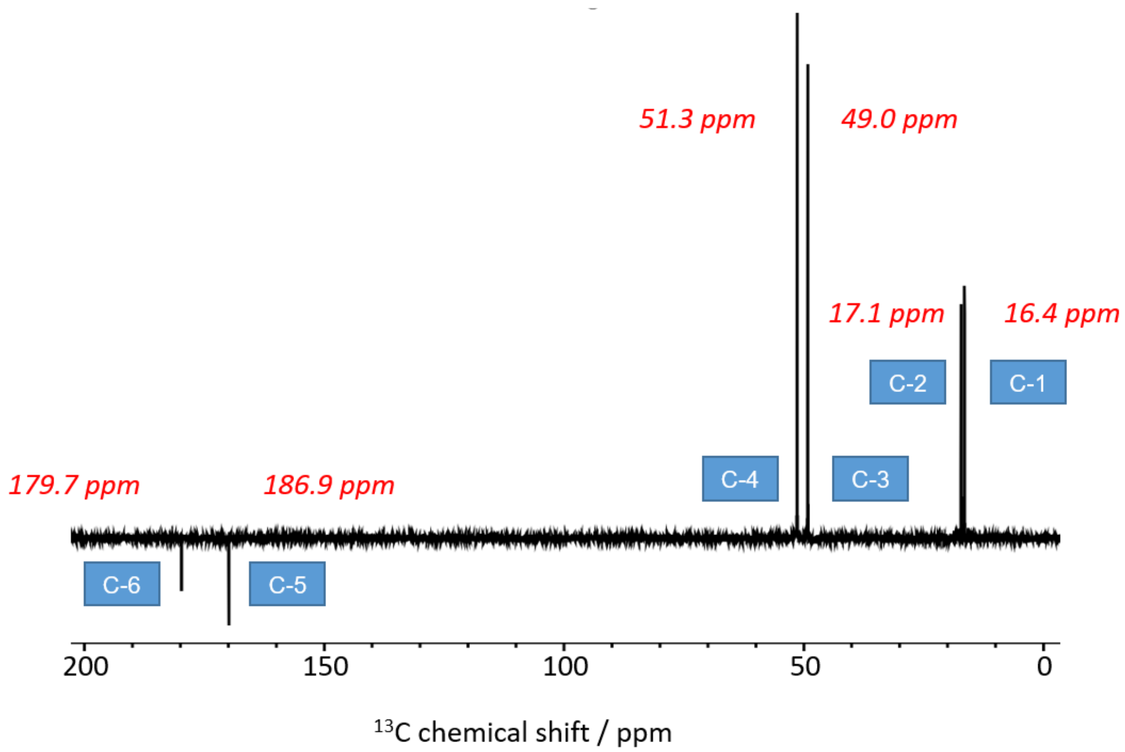

Supplementary Figure 6: ^{13}C-NMR spectrum of 2Ala (D_2O, 120 MHz). The labels of the nuclei are defined in Supplementary Figure 4, ppm values of peaks are shown in red.

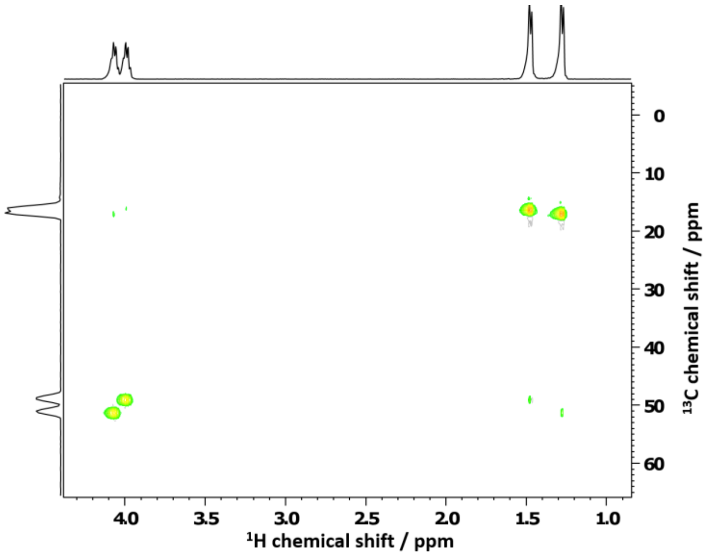

Supplementary Figure 7: ^{1}H-^{13}C-HSQC spectrum of 2Ala (D_2O, 400 MHz / 120 MHz).

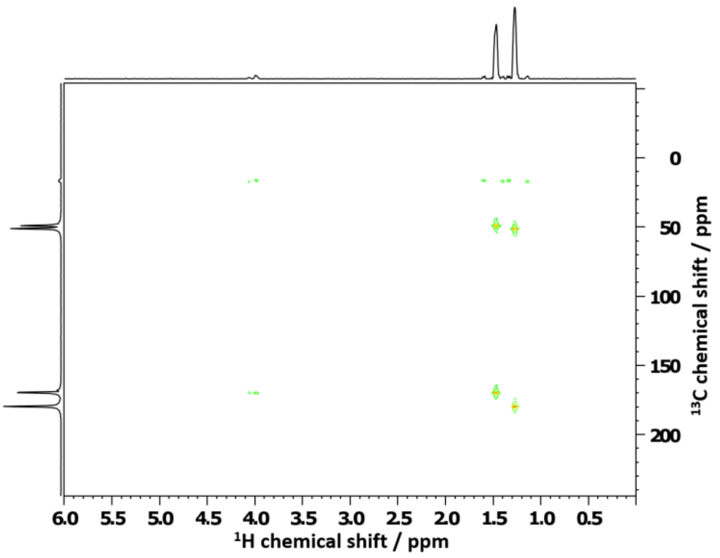

Supplementary Figure 8: ^{1}H-^{13}C-HMBC spectrum of 2Ala (D_2O, 400 MHz, 120 MHz).

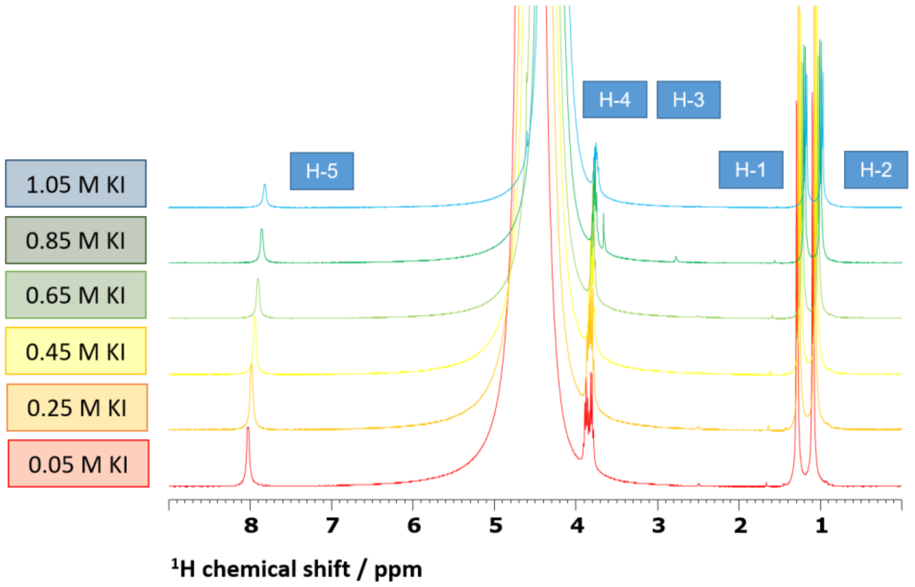

Supplementary Figure 9: ^{1}H-NMR spectra of 2Ala-KI solutions: Also the dominant water signal at about 4.5 ppm undergoes a salt-dependent up-field shift.

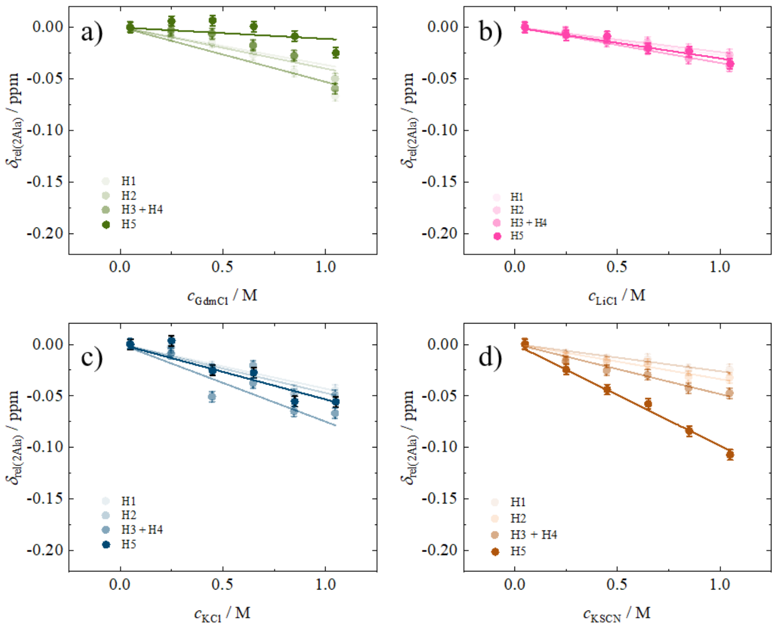

Supplementary Figure 10: Variation of the ^{1}H-NMR chemical shift of all detected 2Ala proton signals for 250 mM 2Ala solutions upon addition of a) GdmCl, b) LiCl, c) KCl, and d) KSCN. Error bars are based on the typical experimental reproducibility (± 0.005 ppm), see main text.

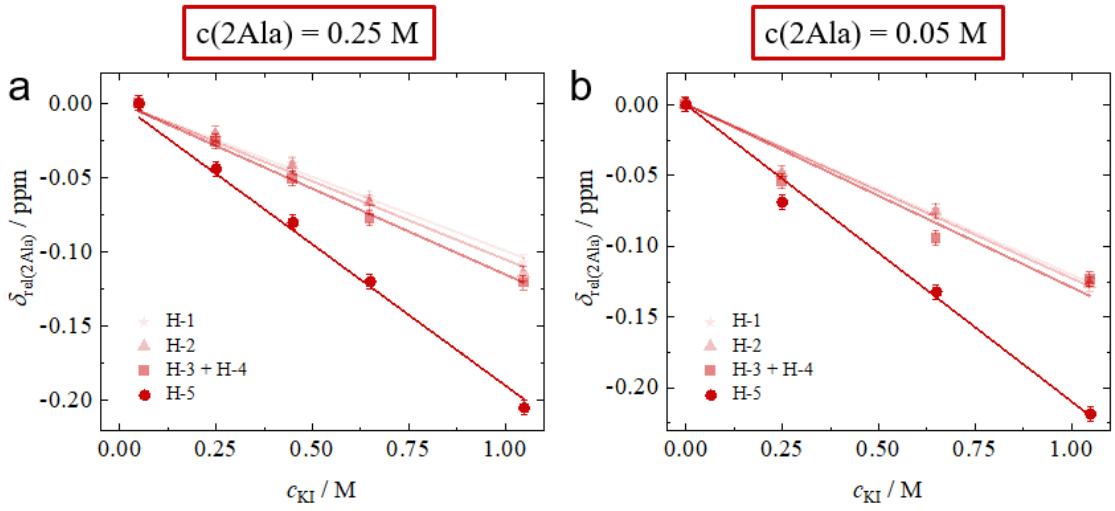

Supplementary Figure 11: Concentration-dependent relative chemical shift for aqueous solutions of a) 250 mM 2Ala and b) 50 mM 2Ala with increasing concentration of KI. Error bars are based on the typical experimental reproducibility (± 0.005 ppm), see main text..

Supplementary Discussion 3: Linear Infrared Spectroscopy

Additional infrared spectra

As discussed in the main manuscript, the presence of the antisymmetric CN stretching mode of Gdm^+ at ~1610 cm^{-1} prevents us from isolating the carbonyl and carboxylate bands for solutions containing GdmCl (Supplementary Supplementary Figure S12). Nevertheless, comparison of the amide I mode (~1670 cm^{-1}) in the presence of different salts at 1 M suggests that this vibrational band is very similar for all studied salts (Supplementary Supplementary Figure S12): Salts at 1 M have a negligible effect on the vibrational properties of 2Ala. Only addition of LiCl results in a moderate broadening of the COO^- band (Supplementary Figure 13). This broadening is also similarly observed at 250 mM 2Ala and 50mM 2Ala, which suggests that aggregation of 2Ala does not affect our observations (Supplementary Figure 14).

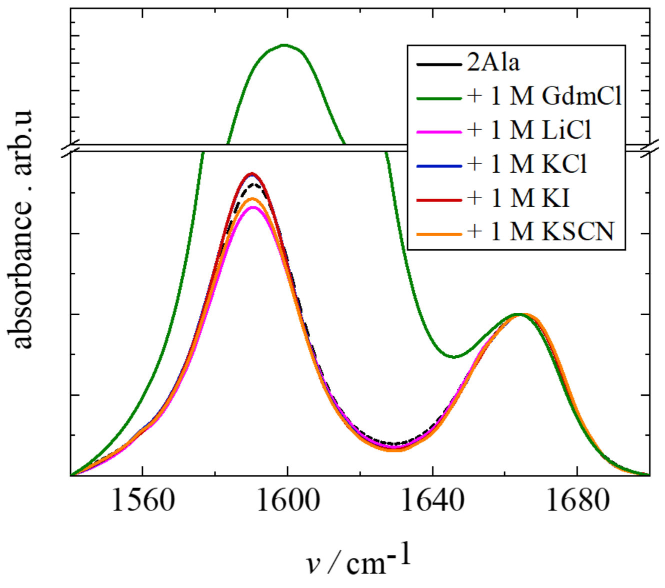

Supplementary Figure S12: Infrared absorption spectra of 1 M salt solutions with 250mM 2Ala. LiCl, KCl, KI, and KSCN hardly affect the IR spectra. The presence of the antisymmetric CN stretching mode of Gdm^+ at ~1610 cm^{-1} [9] prevents us from isolating the carbonyl and carboxylate bands for solutions containing GdmCl. Yet, the blue-wing of the amide I mode with added GdmCl coincides with the wing of the other samples, suggesting that also GdmCl does not affect the amide CO band significantly. Spectra were corrected for a linear background and normalized to the CO peak at ~ 1660 cm^{-1}.

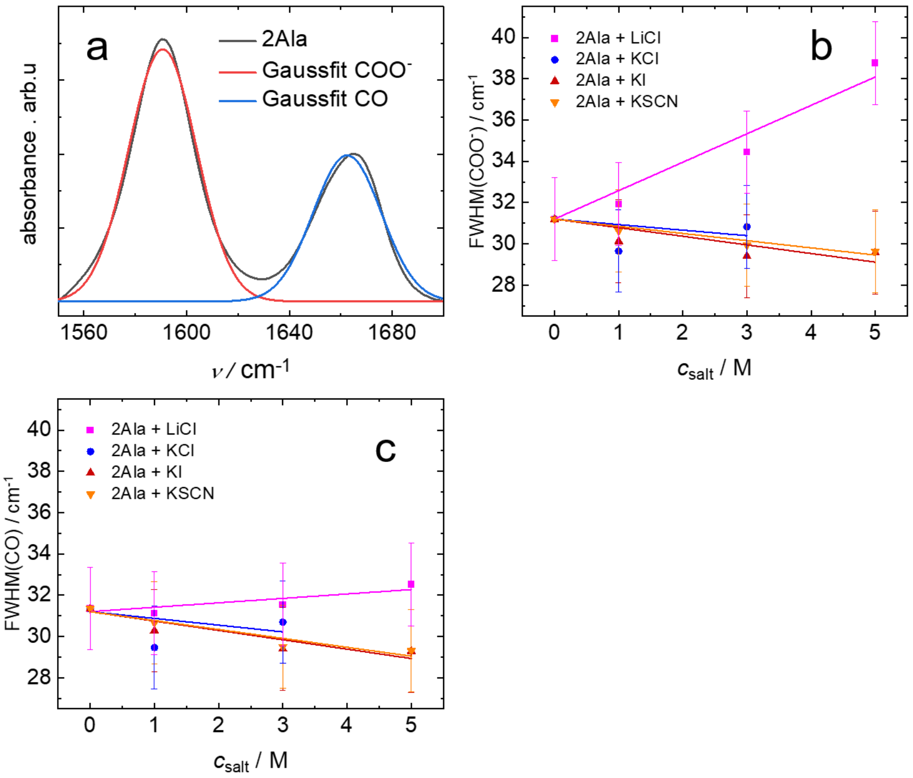

Supplementary Figure S13: a) Linear IR spectrum of 2Ala fitted with two Gaussian functions: Red for the COO^- mode at ~1590 cm^{-1} and blue for the CO mode at ~1660 cm^{-1}. Salt and concentration dependent FWHM of COO^- mode (b) and CO mode (c). Error bars in panel b and c represent the resolution of the spectrometer (4 cm^{-1}).

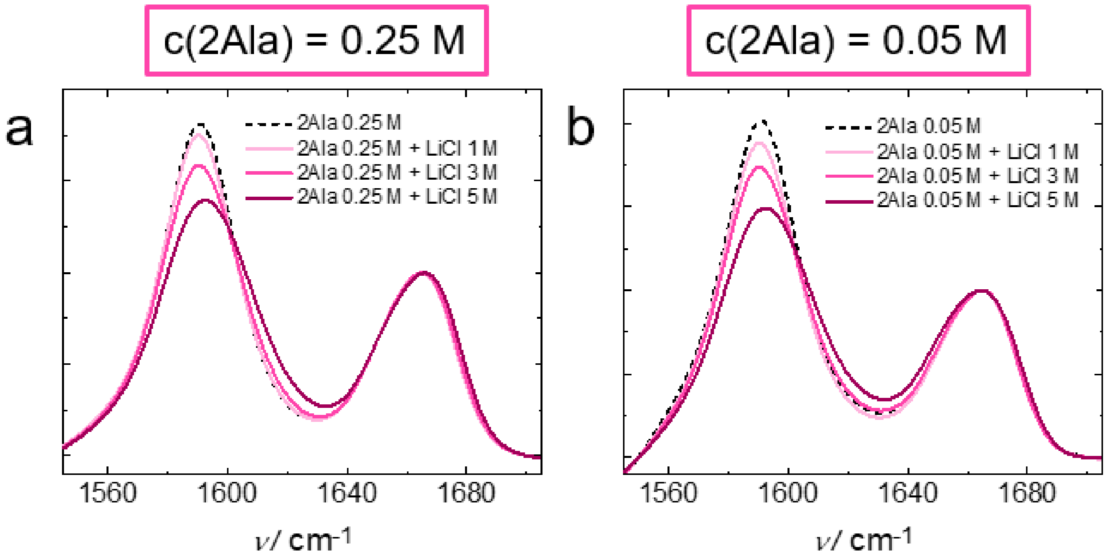

Supplementary Figure 14: Infrared absorption spectra at amide I and asymmetric carboxylate stretching frequencies for solutions of a) 0.25 M 2Ala and b) 0.05 M 2Ala in D_2O with increasing concentration of LiCl. Spectra were normalized to the amide I mode at ~1650 cm^{-1} after subtraction of a linear solvent background. The resemblance of the spectra in panels a) and b) demonstrates that a potential salt induced aggregation of 2Ala does not affect the line shape.

Supplementary Discussion 4: Additional 2D-IR Data and Analysis

Vibrational energy relaxation

To determine the vibrational lifetime of the COO^- and CO modes, we integrate the peak areas of the 2D IR spectra. We use elliptical integration boundaries, as shown in Supplementary Supplementary Figure 15b. We fit a single exponential decay (A_0 exp($-T_2/\tau_{VER}$) + y_0) to these integrals to obtain the characteristic vibrational energy relaxation times (Supplementary Supplementary Figure 15). The lifetime of the two modes is not significantly affected by the addition of salt and remains virtually constant at ~0.7 ps for the CO stretching vibration and at ~0.4 ps for the COO^- asymmetric stretching vibration (Supplementary Supplementary Figure 17).

Analysis of the cross-peak between the amide I and the antisymmetric carboxylate stretching mode

In the main manuscript we show in Figure 7 b the appearance of a cross peak between the amide I and the COO^- mode at later waiting times. As can be seen from the integrated peak volumes shown in Supplementary Supplementary Figure 16, the cross-peak has its maximum intensity at a waiting time of ~200 fs, indicative of energy transfer from the amide I mode to the low frequency carboxylate mode. These dynamics are rather insensitive to the addition of 5 M LiCl. Also the intensity of the cross-peaks, relative to the signal intensity of the diagonal signal of the amide I mode for 2Ala in water and 2Ala + 5 M LiCl are virtually the same, which suggests that addition of LiCl hardly affects the coupling strength of both vibrations. As such, our data suggest that, despite LiCl alters the vibrational structure and dynamics of the COO^- mode (as discussed in the main manuscript), the coupling strength to the amide I mode remains unaffected. This may be due to a cancellation of altered coupling due to a simultaneous variation of the transition dipole strength, transition dipole orientation, and resonance frequency of the COO^- mode upon interaction with Li^+.

Center line slopes

To determine the center line slopes (CLS), we take slices parallel to the probe axis at frequency ranges 1575 – 1610 cm^{-1} for the COO^- mode and at 1640 – 1675 cm^{-1} for the CO mode. We determine the minima of the bleaching signal at a given pump frequency. Such, instrument noise due to pulse-to-pulse energy fluctuations of the probe pulse, which can lead to a distortion of the line shapes parallel to the probe axis, do not affect the position of the determined minima, as the probe pulse energy is constant at a given pump-frequency. We fit these minima linearly and the slope of these linear fits (relative to the pump axis) equals to one if the fit lies on the diagonal of a 2D IR spectrum, while it equals 0 if it is parallel to the pump axis. To determine the rate constant, k, of the decay of the center line slopes (CLS), the CLS was extracted from the 2D spectrum as a function of waiting time (T_2). The 2D-IR spectra used for this analysis are displayed in (Supplementary Supplementary Figure 18 - Supplementary Supplementary Figure *29*). The CLS decays were fit with an exponential decay function ($CLS(T_2)= CLS_0$ exp($-k_j T_2$)). Such fits for the CLS decay of the carboxylate mode at different concentrations of

LiCl are displayed in Supplementary Supplementary Figure 30. The CLS decays for different salts at the highest salt concentration of the present study for both modes are displayed Supplementary Supplementary Figure 31.

We note that a single exponential decay does not model all features of the CLS dynamics and the data in Supplementary Supplementary Figure 31b suggest the presence of oscillatory dynamics, in addition to the decay dynamics. Such oscillatory dynamics indicate that the amide I mode is coupled to a lower frequency vibrational mode, similar to what has been found for water.[10] Interestingly, this coupling is absent for N-methylacetamide, in line with earlier work,[11] and is also hardly detectable for 2Ala at pD 1, at which the carboxylate group is protonated (Supplementary Supplementary Figure 32). This comparison suggests that the coupling to the lower frequency vibration is intimately connected to the presence of the carboxylate group. Yet, due to the limited time window (given by the vibrational energy relaxation time) and the insensitivity of the CLS dynamics of the amide I mode to the addition of salts, we refrain from a more quantitative analysis of this oscillatory feature.

To estimate the error of the parameters obtained from the exponential fits to the CLS decays, we analyze the CLS decay using different frequency ranges (see Supplementary Supplementary Figure 33). For the CLS decay of the COO^- mode, we find that the variation of the decay rate for different frequency ranges is about three times higher than the error as determined from the square root of the diagonal elements of the covariance matrix of the exponential fit to only a single trace as shown in Supplementary Supplementary Figure 33. As such, we estimate the error in the decay rate k to be the triple fitting error of the exponential fit.

The thus determined concentration and salt dependent decay rates for the COO^- and the CO mode are displayed in Supplementary Supplementary Figure 34. The decay rates k are slowed down by the addition of salt in the case of the carboxylate mode (a), while the addition of salt does not have a pronounced effect on the decay rates of the amide I mode (b).

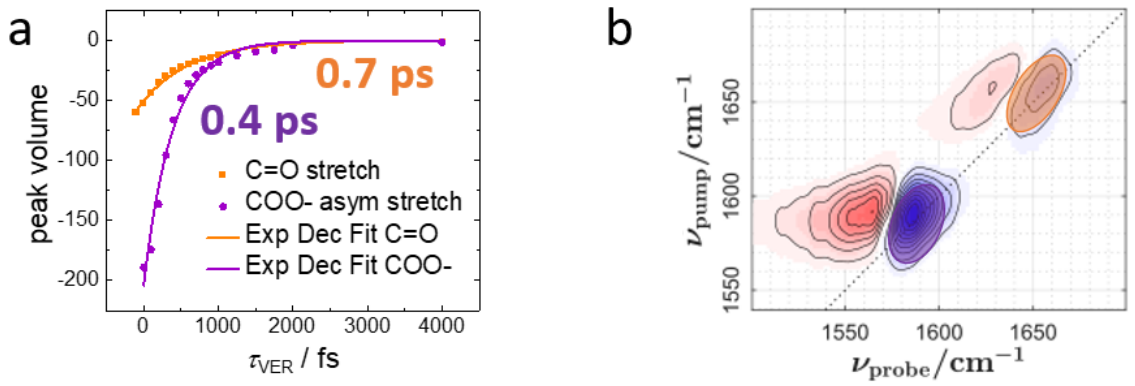

Supplementary Figure 15: a) Waiting time dependent peak volume for 250 mM 2Ala in D_2O for the amide I CO stretch vibration (orange symbols) and for the anti-symmetric COO^- stretching vibration (purple symbols). Solid lines show exponential decay fits to the data. b) The elliptical integration boundaries used for obtaining the peak volumes in panel a) are exemplarily shown at 0 fs waiting time in panel b). These integration boundaries were used at all waiting times.

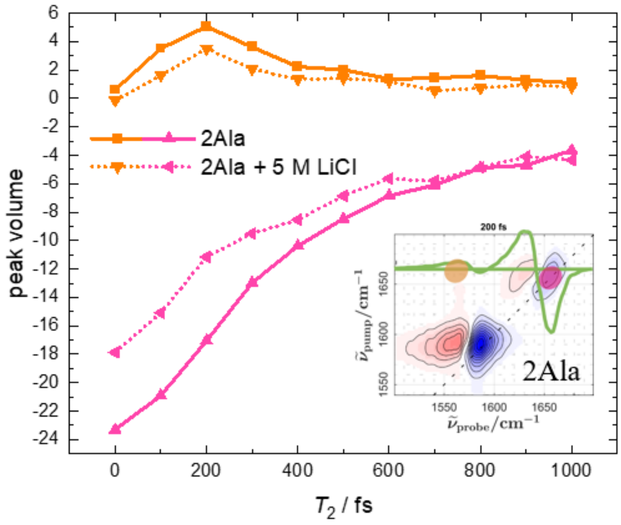

Supplementary Figure 16: Integrated peak volumes of the marked areas of the 2D-IR spectrum of 2Ala (see inset) as a function of waiting time for 2Ala (solid lines) and 2Ala+5M LiCl (dotted line). The pink symbols show the volume of the bleaching signal at the diagonal, orange symbols the evolution of the cross peak.

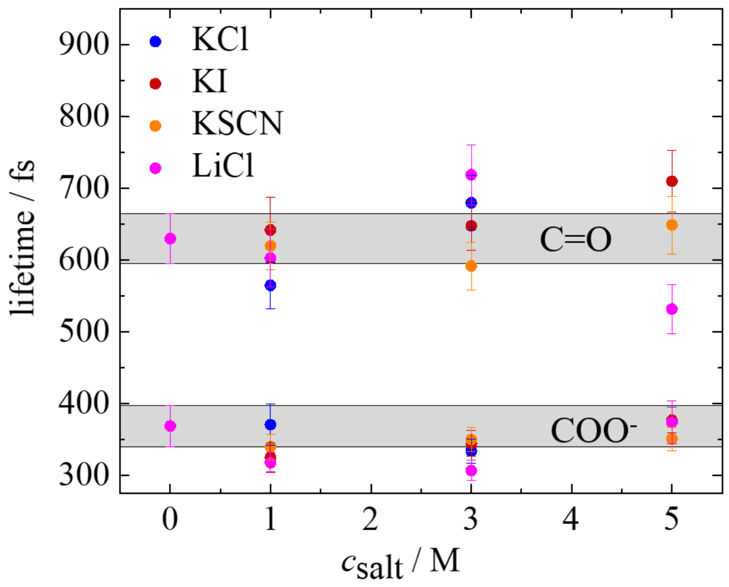

Supplementary Figure 17: Salt and concentration dependent vibrational energy relaxation time for the CO and COO^- modes of 2Ala. The addition of salt does not alter the lifetime of the oscillators significantly. Error bars stem from the error of the fitting parameter of the exponential fit.

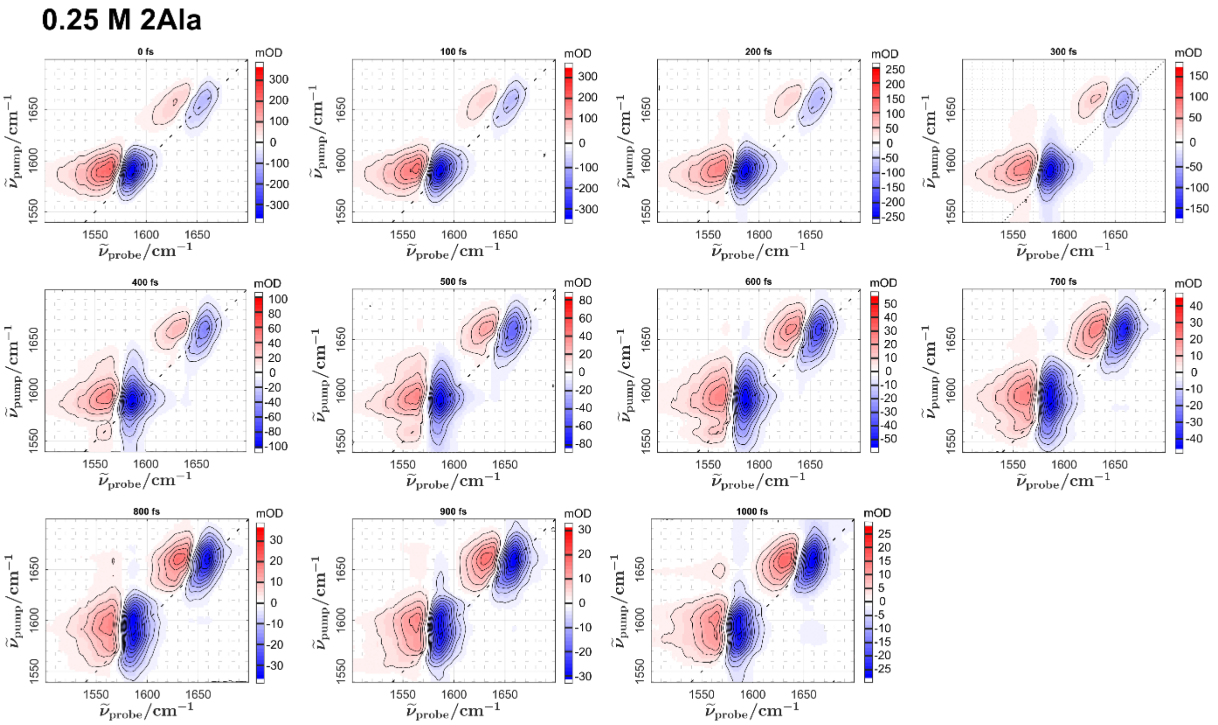

Supplementary Figure 18: 2D IR spectra of 0.25 M 2Ala in D_2O at waiting times ranging from 0 to 1000 fs at increments of 100 fs. All spectra are normalized to their maximum intensity. Scale bars providing the intensities are shown for each spectrum.

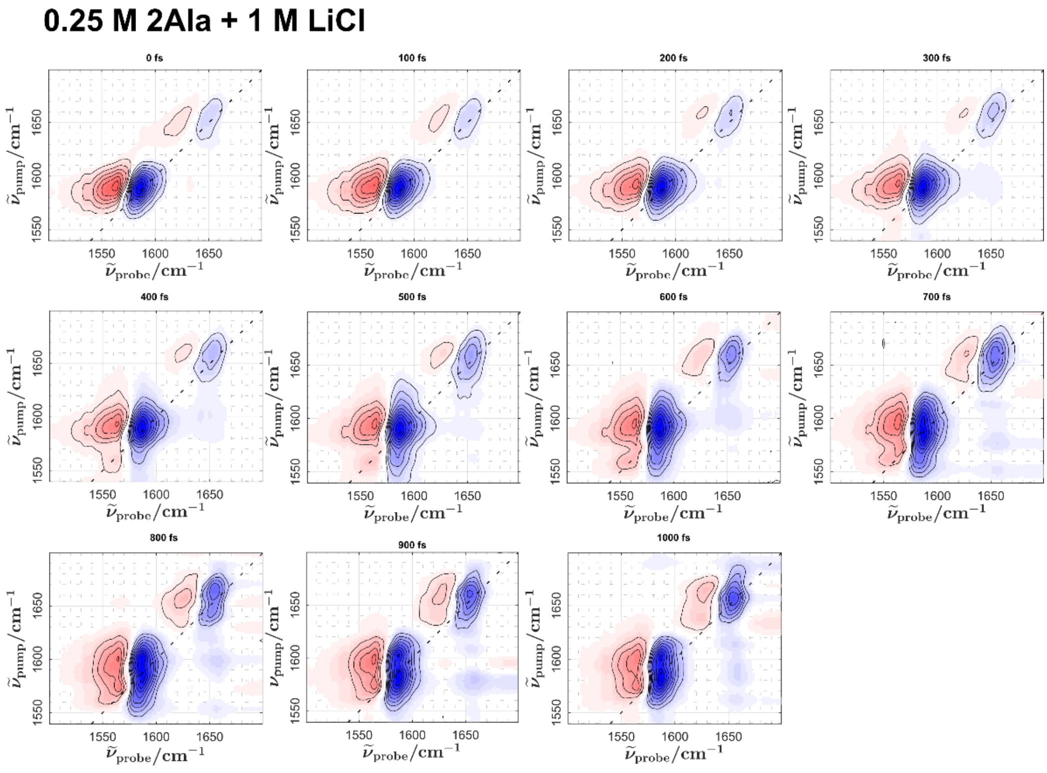

Supplementary Figure 19: 2D IR spectra of 0.25 M 2Ala + 1 M LiCl in D_2O at waiting times ranging from 0 to 1000 fs at increments of 100 fs. All spectra are normalized to their maximum intensity.

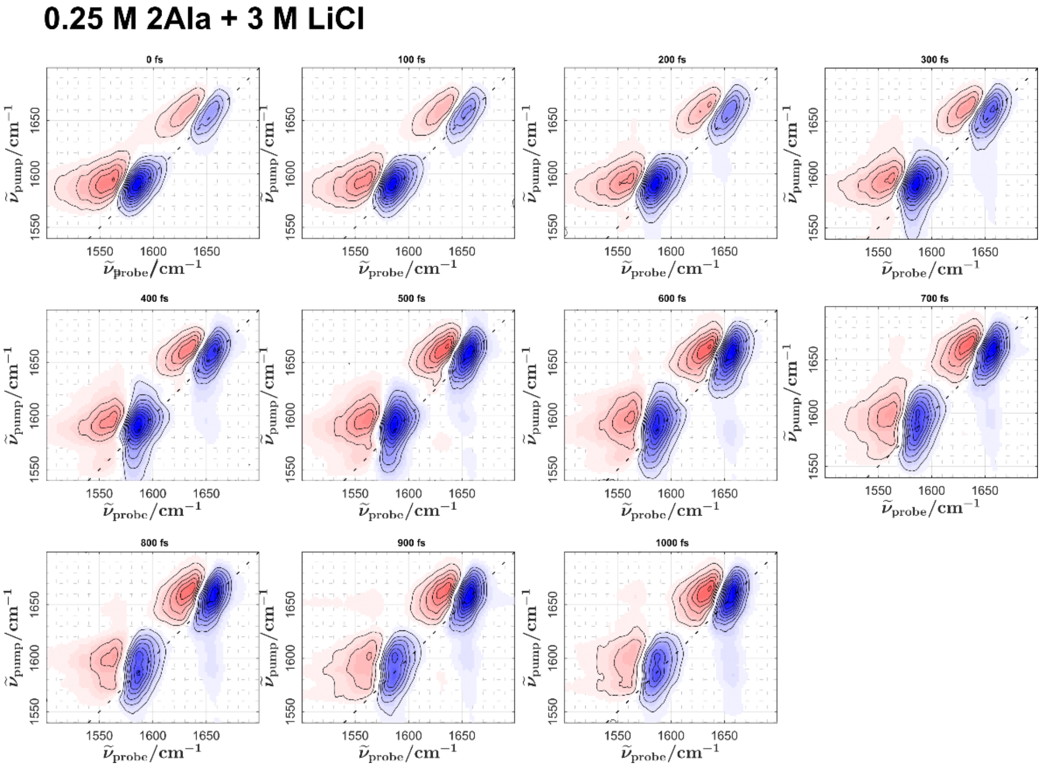

Supplementary Figure 20: 2D IR spectra of 0.25 M 2Ala + 3 M LiCl in D_2O at waiting times ranging from 0 to 1000 fs at increments of 100 fs. All spectra are normalized to their maximum intensity.

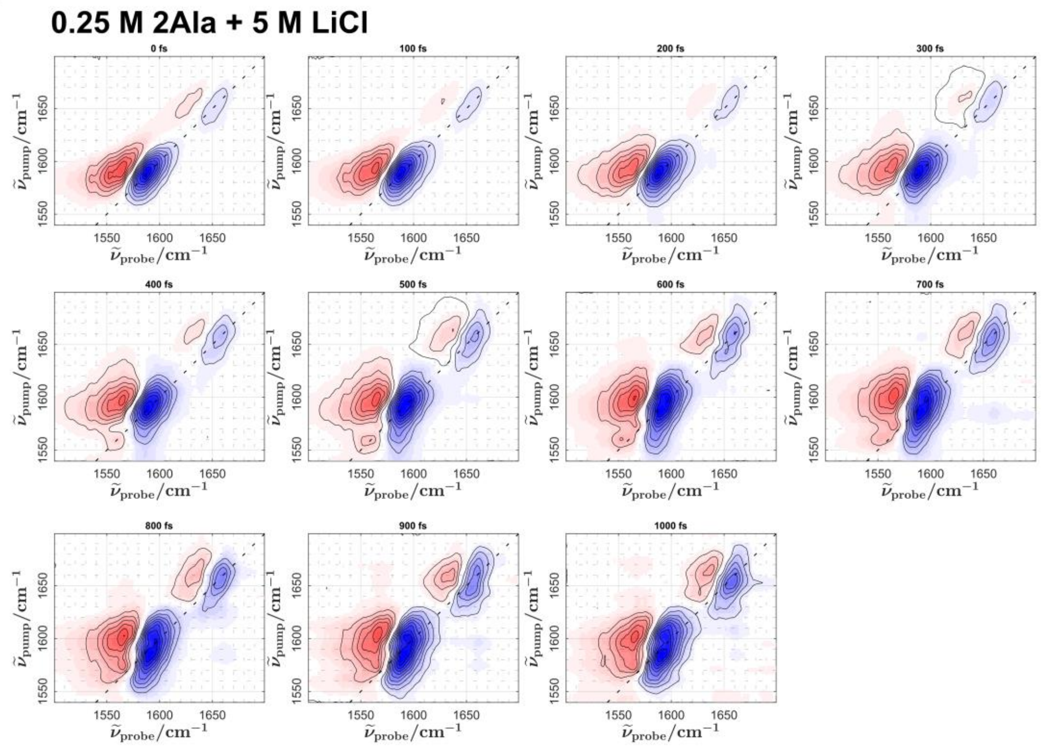

Supplementary Figure 21: 2D IR spectra of 0.25 M 2Ala + 5 M LiCl in D_2O at waiting times ranging from 0 to 1000 fs at increments of 100 fs. All spectra are normalized to their maximum intensity.

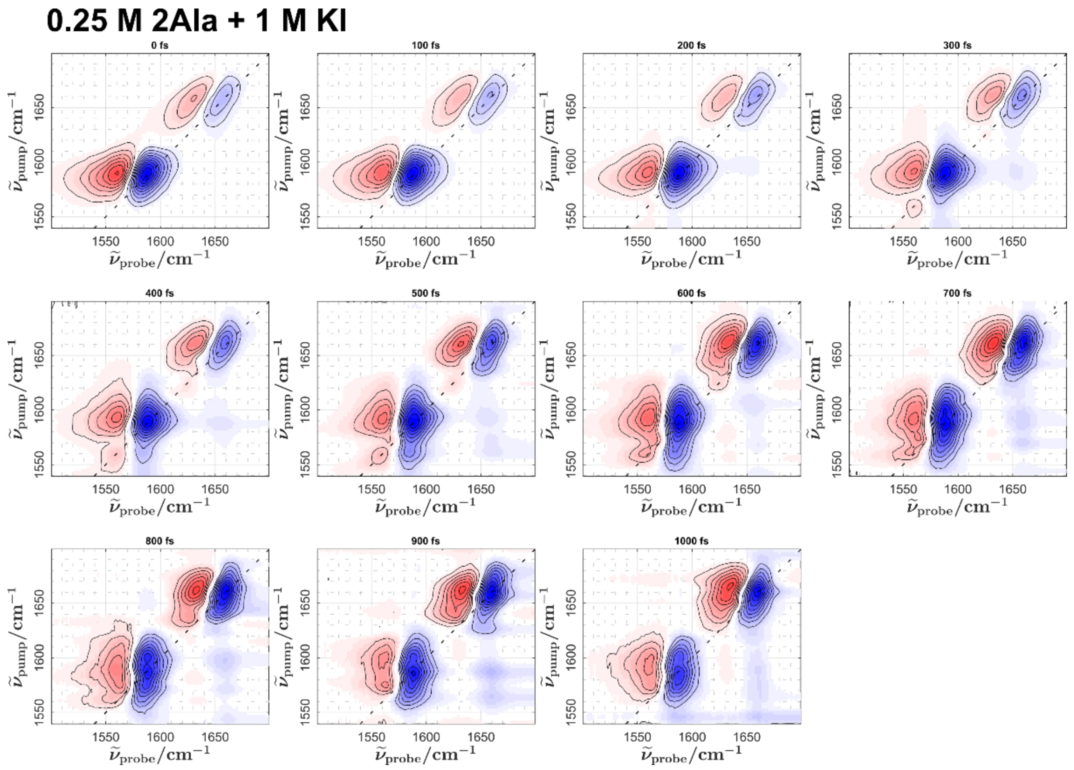

Supplementary Figure 22: 2D IR spectra of 0.25 M 2Ala + 1 M KI in D_2O at waiting times ranging from 0 to 1000 fs at increments of 100 fs. All spectra are normalized to their maximum intensity.

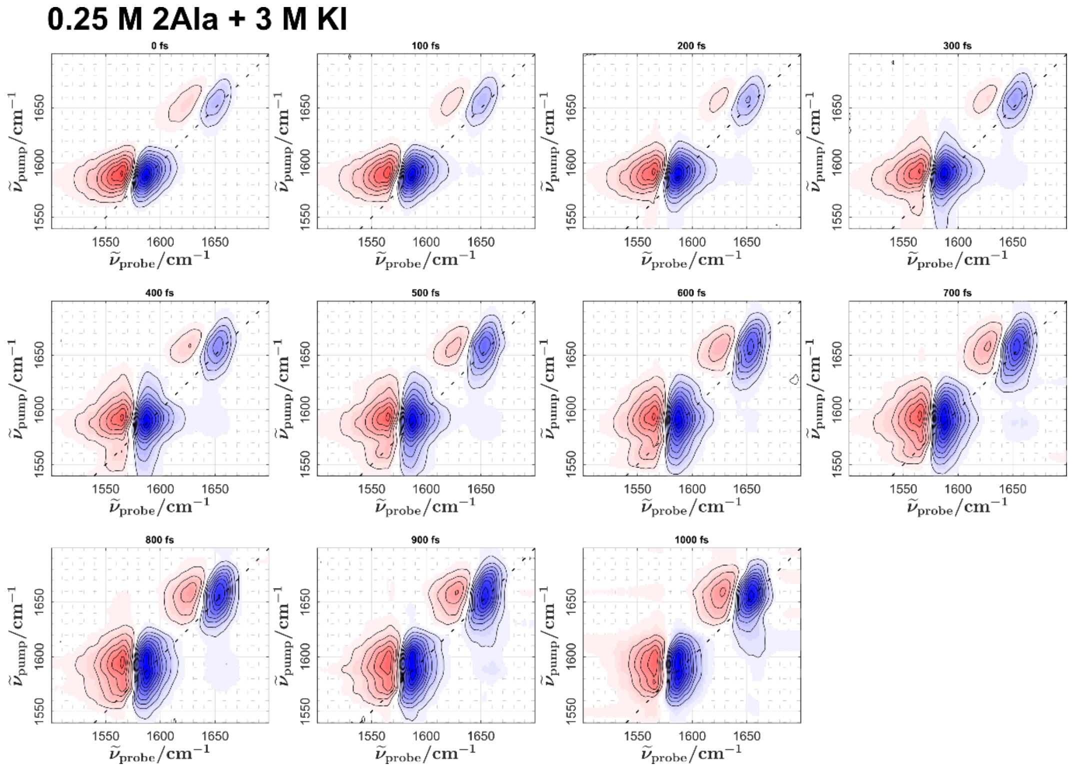

Supplementary Figure 23: 2D IR spectra of 0.25 M 2Ala + 3 M KI in D_2O at waiting times ranging from 0 to 1000 fs at increments of 100 fs. All spectra are normalized to their maximum intensity.

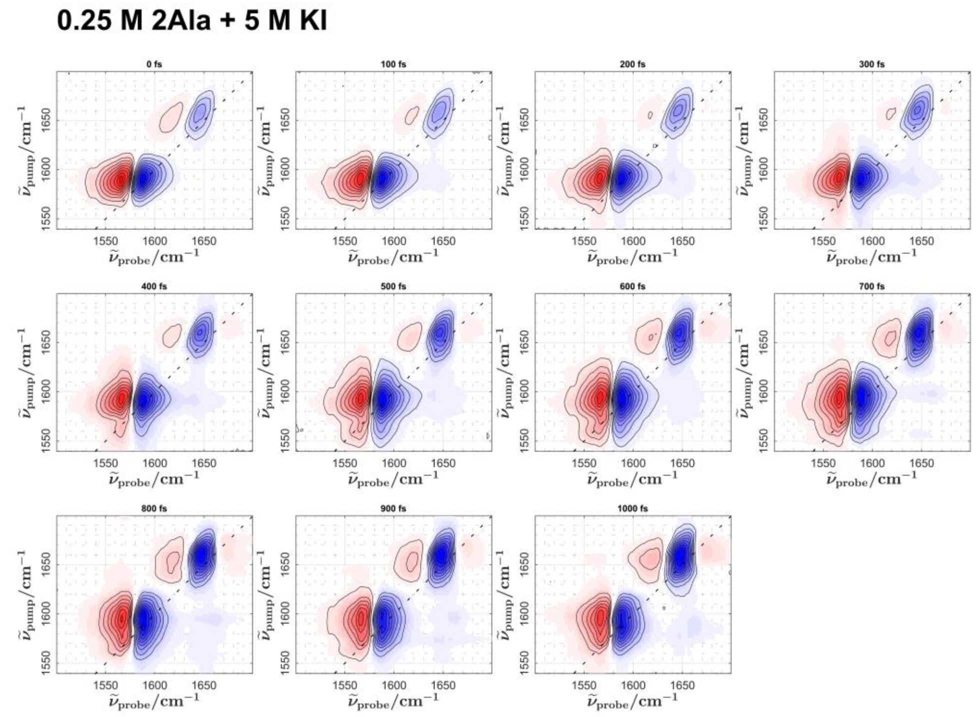

Supplementary Figure 24: 2D IR spectra of 0.25 M 2Ala + 5 M KI in D_2O at waiting times ranging from 0 to 1000 fs at increments of 100 fs. All spectra are normalized to their maximum intensity.

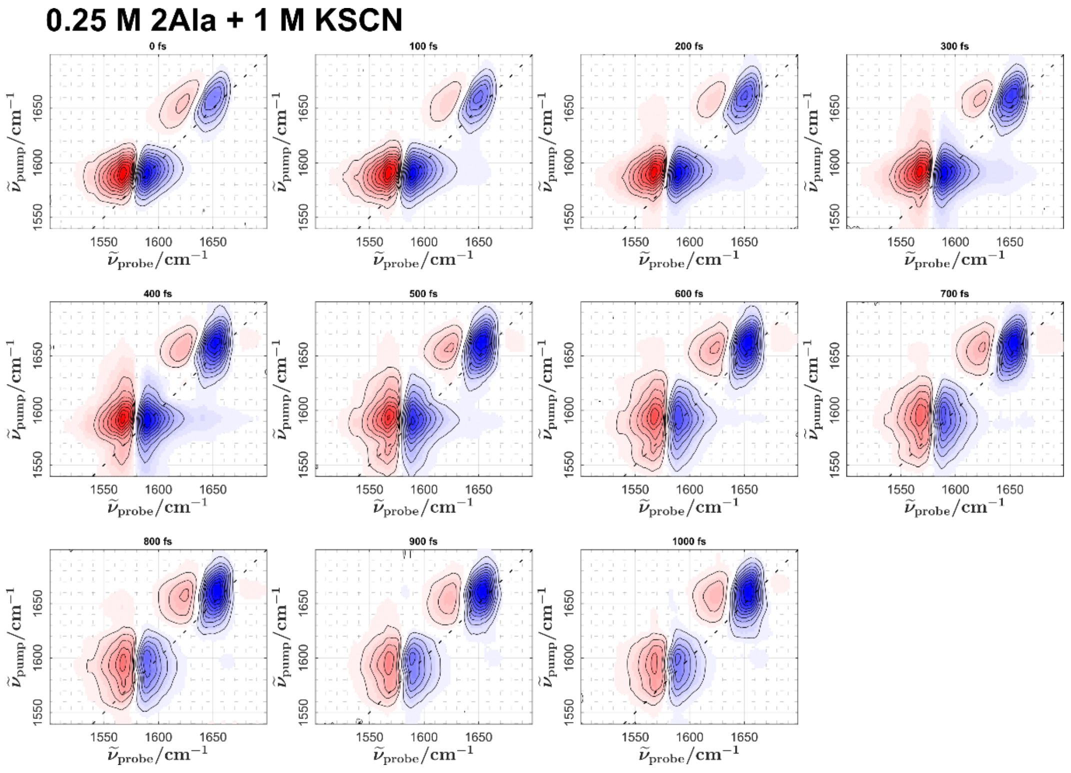

Supplementary Figure 25: 2D IR spectra of 0.25 M 2Ala + 1 M KSCN in D_2O at waiting times ranging from 0 to 1000 fs at increments of 100 fs. All spectra are normalized to their maximum intensity.

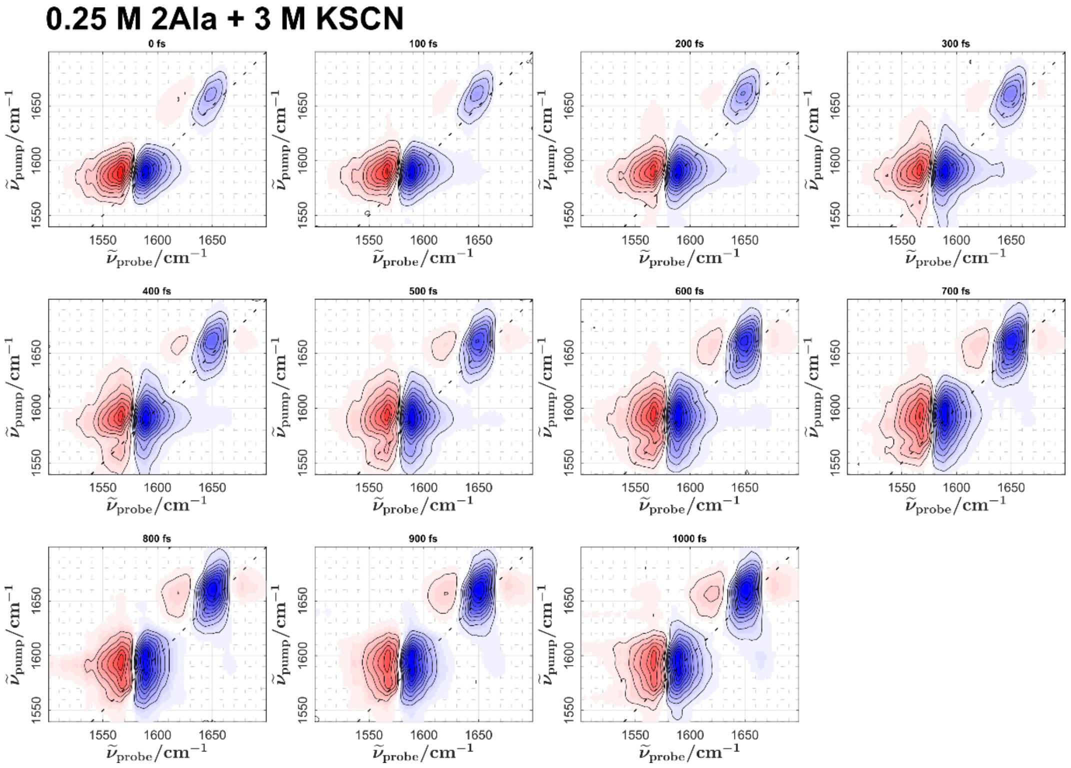

Supplementary Figure 26: 2D IR spectra of 0.25 M 2Al + 3 M KSCN in D_2O at waiting times ranging from 0 to 1000 fs at increments of 100 fs. All spectra are normalized to their maximum intensity.

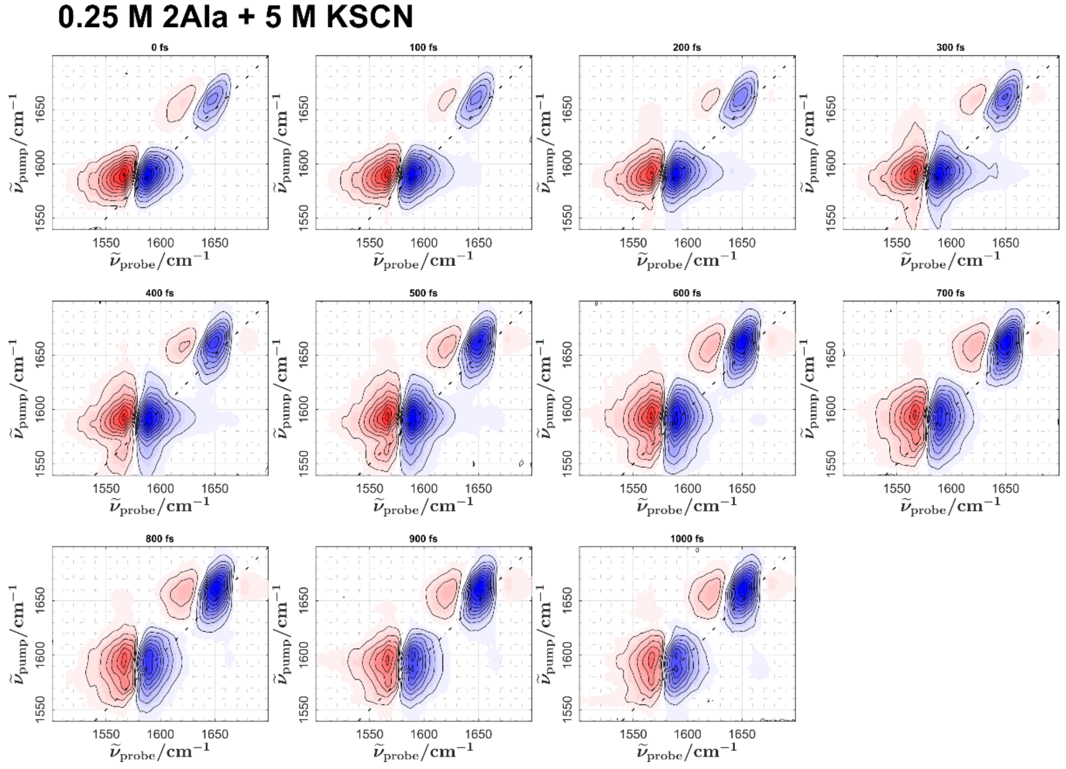

Supplementary Figure 27: 2D IR spectra of 0.25 M 2Ala + 5 M KSCN in D_2O at waiting times ranging from 0 to 1000 fs at increments of 100 fs. All spectra are normalized to their maximum intensity.

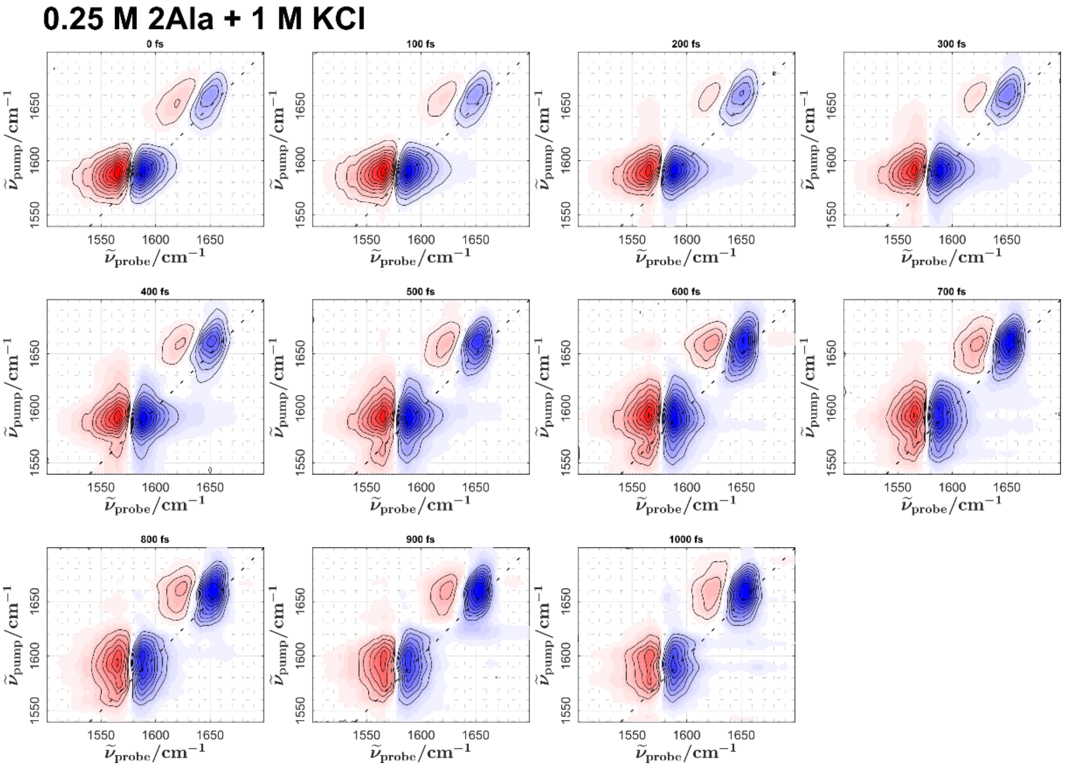

Supplementary Figure 28: 2D IR spectra of 0.25 M 2Ala + 1 M KCl in at waiting times ranging from 0 to 1000 fs at increments of 100 fs. All spectra are normalized to their maximum intensity.

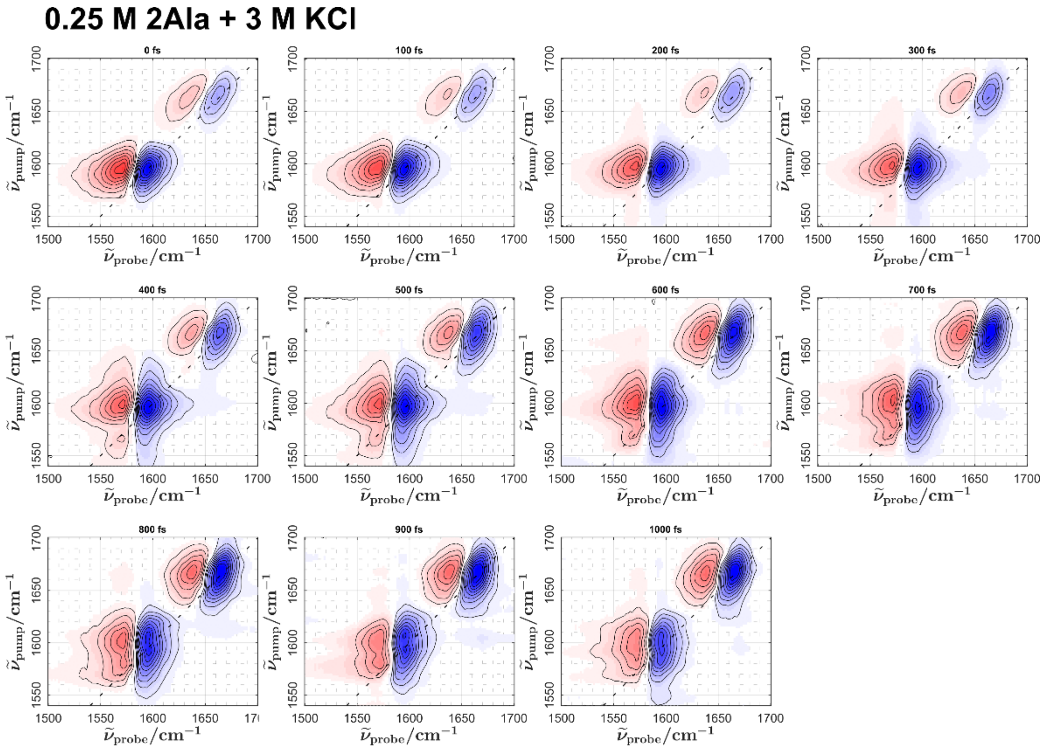

Supplementary Figure 29: 2D IR spectra of 0.25 M 2Ala + 3 M KCl in D_2O at waiting times ranging from 0 to 1000 fs at increments of 100 fs. All spectra are normalized to their maximum intensity

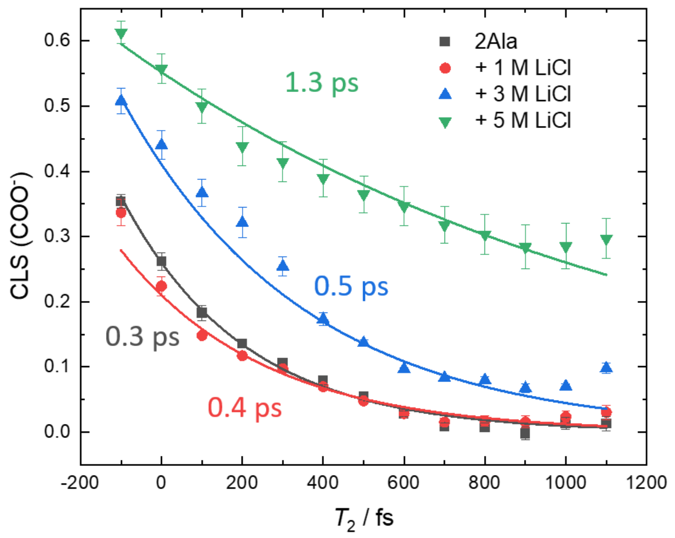

Supplementary Figure 30: CLS (center line slope) decay of the carboxylate mode for solutions of 250 mM 2Ala in D_2O in the presence of different concentrations of LiCl. Symbols show the extracted CLS data and solid lines show exponential fits. The decay time 1/k is indicated in the figure. Error bars correspond to the standard deviation of the CLS of the bleaching signal determined from five different scans.

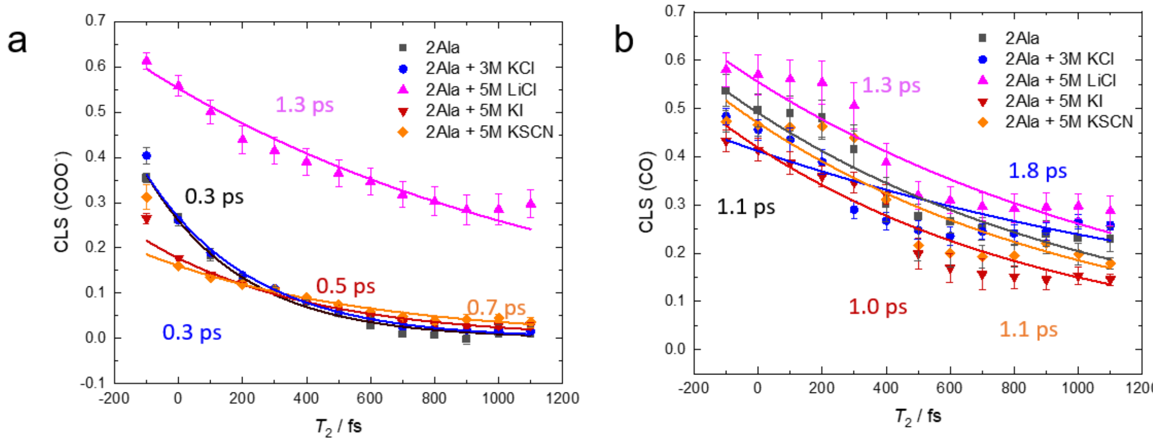

Supplementary Figure 31: CLS (center line slope) decays for (a) the carboxylate mode and (b) the amide I mode for pure 2Ala solutions and in the presence of 3 M KCl, 5 M LiCl, 5 M KI, and 5 M KSCN. Symbols show the extracted CLS data, and solid lines show exponential fits. The decay time 1/k is indicated in the figure. Error bars correspond to the standard deviation of the CLS of the bleaching signal determined from five different scans.

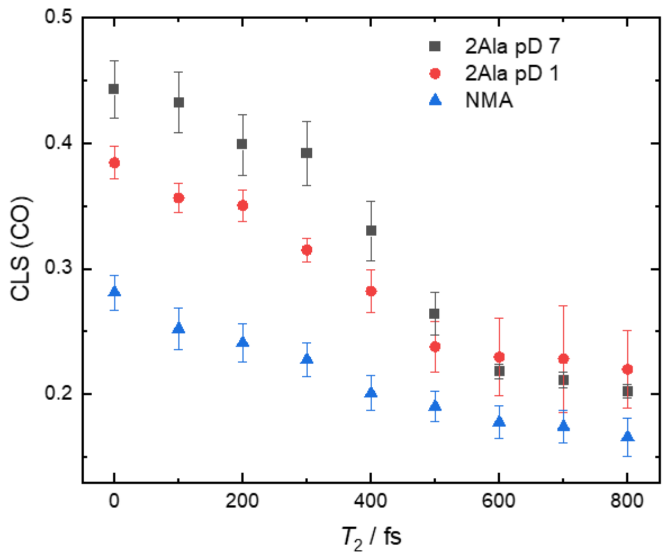

Supplementary Figure 32: Center line slopes of the amide I mode of 2Ala at pD 7, 2Ala at pD 1, and N-methylacetamide (NMA) dissolved in D_2O as a function of waiting time. Error bars correspond to the standard deviation of the CLS of the bleaching signal determined from five different scans.

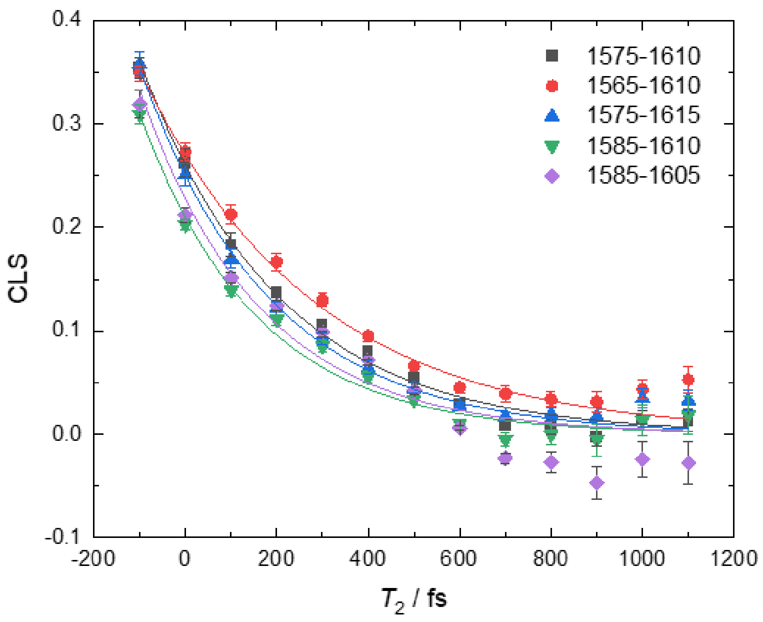

Supplementary Figure 33: Waiting time dependent CLS (center line slope) of the COO^- mode for pure 2Ala in D_2O with the CLS determined over different frequency ranges as indicated in the legend. Symbols show extracted CLS values. Error bars correspond to the standard deviation of the CLS of the bleaching signal determined from five different scans.

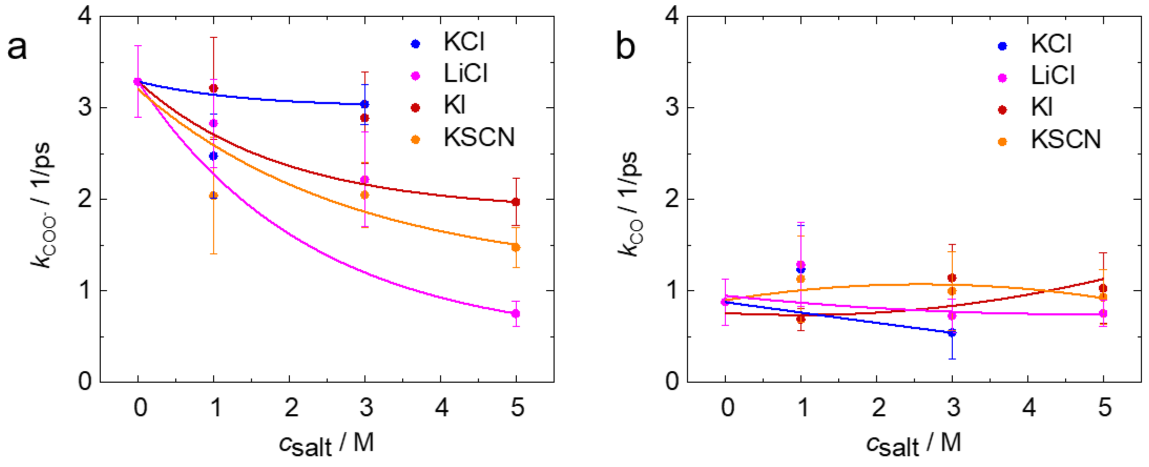

Supplementary Figure 34: The waiting-time-dependent (T_2) CLS (center line slope) decay rates as determined from single exponential fits ($CLS(T_2)= CLS0\ exp(-k_j\ T_2)$) for a) COO^-. ($j = COO^-$) and b) CO (j = CO). Symbols are experimental data and solid lines are a visual aid. Error bars correspond to the triple standard deviation of the CLS determined from five different frequency ranges (see Supplementary Supplementary Figure 33).

Supplementary References

1. Kremer, F. & Schönhals, A. *Broadband Dielectric Spectroscopy*. (Springer-Verlag Berlin Heidelberg GmbH, Berlin, 2003).

2. Sato, T., Buchner, R., Fernandez, Š., Chiba, A. & Kunz, W. Dielectric relaxation spectroscopy of aqueous amino acid solutions: dynamics and interactions in aqueous glycine. *J. Mol. Liq.* **117**, 93–98 (2005).

3. Cavell, E. A. S., Knight, P. C. & A., S. M. Dielectric Relaxation in Non Aqueous Solutions. *Trans. Faraday. Soc* **67**, 2225 (1971).

4. Dote, J. L., Kivelson, D. & Schwartz, R. N. A molecular quasi-hydrodynamic free-space model for molecular rotational relaxation in liquids. *J. Phys. Chem.* **85**, 2169–2180 (1981).

5. Gonçalves, A. D. *et al.* The effect of protein concentration on the viscosity of a recombinant albumin solution formulation. *RSC Adv.* **6**, 15143–15154 (2016).

6. Balos, V. *et al.* Specific Ion Effects on an Oligopeptide: Bidentate Binding Matters for the Guanidinium Cation. *Angew. Chemie - Int. Ed.* **58**, 332–337 (2019).

7. Balos, V., Bonn, M. & Hunger, J. Quantifying transient interactions between amide groups and the guanidinium cation. *Phys. Chem. Chem. Phys.* **17**, 28539–28543 (2015).

8. Balos, V., Bonn, M. & Hunger, J. Anionic and cationic Hofmeister effects are non-additive for guanidinium salts. *Phys. Chem. Chem. Phys.* **19**, 9724–9728 (2017).

9. Drozd, M. Molecular structure and infrared spectra of guanidinium cation. A combined theoretical and spectroscopic study. *Mater. Sci. Eng. B Solid-State Mater. Adv. Technol.* **136**, 20–28 (2007).

10. Fecko, C. J., Eaves, J. D., Loparo, J. J., Tokmakoff, A. & Geissler, P. L. Ultrafast Hydrogen-Bond Dynamics in the Infrared Spectroscopy of Water. *Science* **301**, 1698–1702 (2003).

11. DeCamp, M. F. *et al.* Amide I vibrational dynamics of N-methylacetamide in polar solvents: The role of electrostatic interactions. *J. Phys. Chem. B* **109**, 11016–11026 (2005).
